# Predation of potential insect pests in oil palm plantations, rubber tree plantations, and fruit orchards

**DOI:** 10.1002/ece3.5856

**Published:** 2019-12-31

**Authors:** Nuradilah Denan, Wan Mamat Wan Zaki, Ahmad R. Norhisham, Ruzana Sanusi, Dzulhelmi Muhammad Nasir, Frisco Nobilly, Adham Ashton‐Butt, Alex M. Lechner, Badrul Azhar

**Affiliations:** ^1^ Faculty of Forestry Universiti Putra Malaysia Serdang Malaysia; ^2^ Institute of Tropical Forestry and Forest Product Universiti Putra Malaysia Serdang Malaysia; ^3^ Biological Research Division Malaysian Palm Oil Board 6 Persiaran Institusi Kajang Malaysia; ^4^ Department of Animal Science Faculty of Agriculture Universiti Putra Malaysia Serdang Malaysia; ^5^ Laboratoire d'Excellence (LabEx) Sustainable Tropical Agriculture and Food Systems UPM‐Agropolis International Offshore Office Montpellier France; ^6^ British Trust for Ornithology The Nunnery Thetford UK; ^7^ School of Environmental and Geographical Sciences University of Nottingham Malaysia Semenyih Malaysia; ^8^ Biodiversity Unit Institute of Bioscience Universiti Putra Malaysia Serdang Malaysia

**Keywords:** artificial caterpillar, biodiversity, ecosystem services, monoculture, polyculture

## Abstract

In human‐modified landscapes, important ecological functions such as predation are negatively affected by anthropogenic activities, including the use of pesticides and habitat degradation. Predation of insect pests is an indicator of healthy ecosystem functioning, which provides important ecosystem services, especially for agricultural systems. In this study, we compare predation attempts from arthropods, mammals, and birds on artificial caterpillars in the understory, between three tropical agricultural land‐use types: oil palm plantations, rubber tree plantations, and fruit orchards. We collected a range of local and landscape‐scale data including undergrowth vegetation structure; elevation; proximity to forest; and canopy cover in order to understand how environmental variables can affect predation. In all three land‐use types, our results showed that arthropods and mammals were important predators of artificial caterpillars and there was little predation by birds. We did not find any effect of the environmental variables on predation. There was an interactive effect between land‐use type and predator type. Predation by mammals was considerably higher in fruit orchards and rubber tree than in oil palm plantations, likely due to their ability to support higher abundances of insectivorous mammals. In order to maintain or enhance natural pest control in these common tropical agricultural land‐use types, management practices that benefit insectivorous animals should be introduced, such as the reduction of pesticides, improvement of understory vegetation, and local and landscape heterogeneity.

## Graphical abstract

Arthropods were the most dominant predators, followed by mammals and birds. Predation by mammals was considerably higher in fruit orchards and rubber tree than in oil palm plantations. Natural predators should be promoted as an alternative to chemical insecticides.

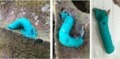

## 1 INTRODUCTION

The contribution of natural enemies of insect pests as biological control agents has increasingly attracted attention, as farmers search for more environmentally friendly alternatives to pesticides (de Groot, Wilson, & Boumans, 2002; Wood, 2002). Pesticides, particularly nonselective ones, have caused extensive loss of biodiversity in agricultural habitats due to their indiscriminate effects on nontarget fauna (Le Roux et al., 2008) and have been blamed for destroying natural pest control services in various agricultural systems. The promotion of natural biological control agents can potentially reduce chemical usage and labor costs in various agricultural systems (Cleveland et al., 2006; Kellermann, Johnson, Stercho, & Hackett, 2008; MEA, 2005; Sekercioglu, 2012).

Local and landscape factors are essential to supporting biological controls (Lindgren, Lindborg, & Cousins, 2018; Milligan, Johnson, Garfinkel, Smith, & Njoroge, 2016; Nurdiansyah, Denmead, Clough, Wiegand, & Tscharntke, 2016; Seifert, Lehner, Adams, & Fiedler, 2015). The presence of biological control agents is strongly affected by vegetation characteristics as heterogeneous vegetation offers a variety of resources and niches (Azhar et al., 2015; Denmead et al., 2017; Nurdiansyah et al., 2016). Similarly, agricultural areas which have greater landscape heterogeneity show higher predation compared with landscapes which are predominantly monocultures (Rusch et al., 2016).

There are a range of pest species in agricultural systems, of which lepidopteran larvae have one of the greatest negative economic impacts in fruit orchards (Elsey & Sirichoti, 2003; García, Miñarro, & Martínez‐Sastre, 2018; Simon, Lesueur‐Jannoyer, Plénet, Lauri, & Bellec, 2017), oil palm plantations (Basri, Norman, & Hamdan, 1995; Corley & Tinker, 2008; Kamarudin & Wahid, 2010; Wood, 2002), and rubber tree plantations (Jayasinghe, 1999; Winder, 1976). In order to assess natural predation of these critical agricultural pests, experiments utilizing artificial caterpillars have been used and represent one of the most robust methodologies for measuring biological pest control (Jedlicka, Greenberg, & Letourneau, 2011). Researchers have recently started using artificial caterpillars as bait and recording predation rates (Howe, Nachman, & Lövei, 2015; Lemessa, Hambäck, & Hylander, 2015; Low, Sam, McArthur, Posa, & Hochuli, 2014; Maas, Tscharntke, Saleh, Dwi Putra, & Clough, 2015; Nurdiansyah et al., 2016; Roels, Porter, & Lindell, 2018; Seifert et al., 2015). Predation pressure and influence of landscape management on pest control can be determined using artificial caterpillar experiment (Low et al., 2014). This method has been tested in a range of latitudes, elevations, and landscape contexts (Howe, Lövei, & Nachman, 2009; Milligan et al., 2016; Nurdiansyah et al., 2016; Roslin et al., 2017). However, most studies in developing countries have so far focused on only a single agricultural crop (i.e., Howe et al., 2009; Koh & Menge, 2006; Nurdiansyah et al., 2016).

This study aims to assess one of the key ecosystem services provided by biodiversity in Southeast Asian agricultural systems. We identified which natural predators are likely to be effective biological control agents in the understory of fruit orchards, oil palm plantations, and rubber tree plantations by assessing attack marks left by predators on artificial caterpillars (Curtis et al., 2013). We examined how local and landscape environmental variables influence predation on artificial caterpillars and assume that predation on pest insects would follow similar patterns. We predicted that fruit orchards, with their increased levels of vegetation heterogeneity and native fruit trees, would experience higher predation than the other land‐use types due to a greater abundance of predators.

## 2 METHODS

### 2.1 Study area

**Figure 1:**
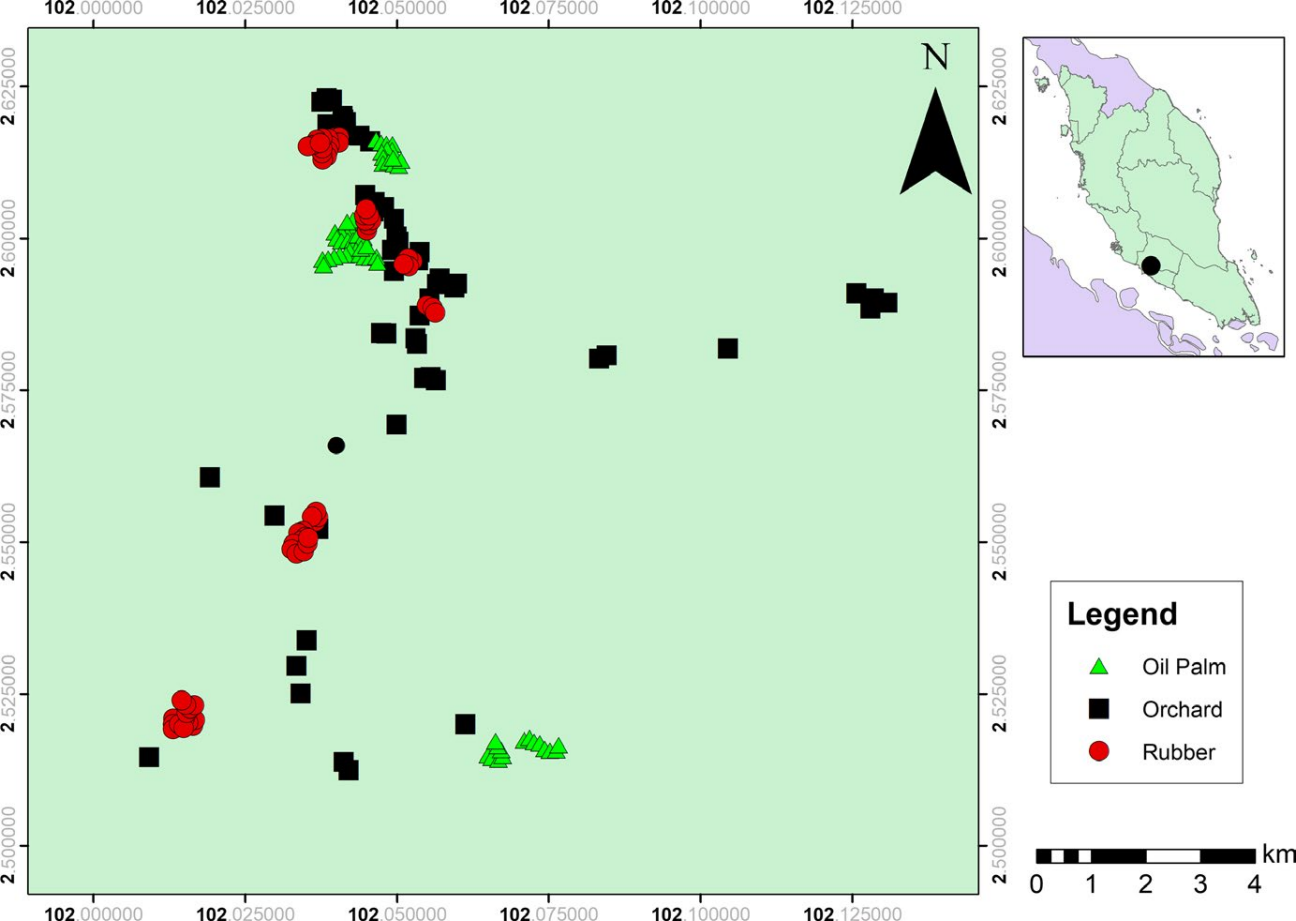
Map of study area shows the plots are located in the state of Negeri Sembilan, Peninsular Malaysia

**Figure 2:**
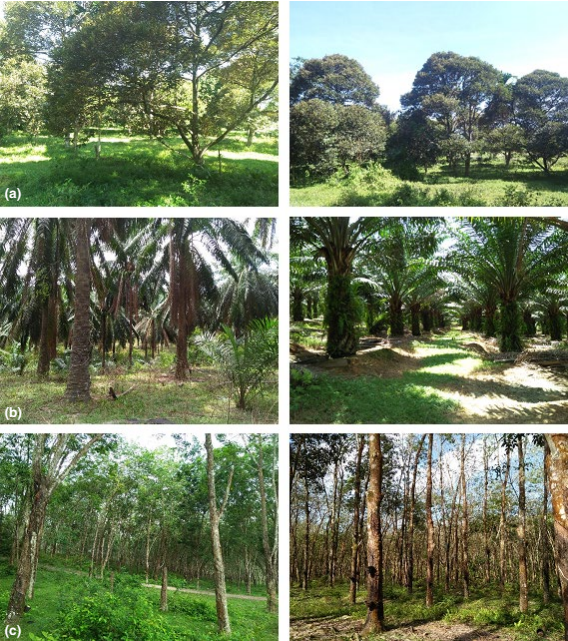
Production landscapes represented by (a) fruit orchard, (b) oil palm plantation, and (c) rubber tree plantation

The study was conducted between Pedas (2°37ʹ13.08″N, 102° 03ʹ27.88″E) and Tampin (2°31ʹ08.35″N, 102°00ʹ55.41″E) in Negeri Sembilan, west coast of Peninsular Malaysia (Figure 1). Data were collected from January to June 2018 during the dry season. The study area was converted at least 60 years ago from lowland dipterocarp forest to agricultural areas. Three agricultural land‐use types were surveyed, consisting of mixed fruit orchards, oil palm plantations, and rubber tree plantations (Figure 2). Fruit orchards were small‐scale and mostly managed by villagers. They were planted with a variety of fruit trees such as Durian (*Durio zibethinus*), Rambutan (*Nephelium* spp.), Jackfruit (*Artocarpus heterophyllus*), Langsat (*Lansium parasiticum*, *Lansium domesticum*), Mangosteen (*Garcinia mangostana*), Papaya (*Carica papaya*), Mango (*Mangifera indica*), and Chempedak (*Artocarpus integer*).

### 2.2 Study design

A total of 180 plots were established across the study site. Sixty individual plots with a 50 m radius were established in each land‐use type (fruit orchards, oil palm plantations, and rubber tree plantations). We used a systematic sampling design with a random start (Morrison, Block, Strickland, Collier, & Peterson, 2008) for each crop, with the next plot at least 100 m away.

### 2.3 Artificial caterpillar preparation, deployment, and identification

Artificial caterpillars were made from nontoxic green‐colored clay plasticine modeling compound, rolled by hand to shape the caterpillars into a cylindrical form, and standardized to 4.5 cm in length and 0.7 cm in width (Howe et al., 2009). The clay contains wheat and is not harmful if consumed by a predator, conforming to ASTM D‐4236(2016) the Standard Practice for Labelling Art Materials for Chronic Health Hazards.

To attach the caterpillars on the vegetation surfaces, adhesive glue was applied at both ends of the caterpillar. Artificial caterpillars were bent in the middle to mimic real caterpillar. Glue use was minimized to avoid any excessive smells. Artificial caterpillars were glued to leaves/fronds, branches of trees, or oil palm trunks and also on understory vegetation which was 0.5 m above ground and at least 5 m apart from each other (Seifert et al., 2015). Five artificial caterpillars were deployed haphazardly within each plot, resulting in a total of 900 artificial caterpillars for the whole study (3 agriculture types × 60 plots × 5 artificial caterpillars). The caterpillars were deployed in each land‐use type simultaneously.

**Figure 3:**
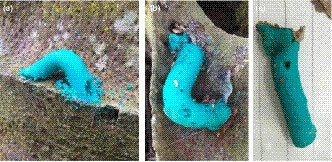
Predators’ attack marks on artificial caterpillar: (a) predation by arthropods (ants), (b) mammal, and (c) bird

The caterpillars were left exposed to predation for 72 hr. On the third day, an inspection was carried out with the aid of a magnifying glass to examine the visible attack marks left by predators (Figure 3). We identified the animal taxon which caused the attack marks. We counted the number of attacks based on the multiple attack marks left on artificial caterpillars.

Any visible marks observed on artificial caterpillar were considered evidence of predation (Low et al., 2014). Identified predator marks were analyzed based on the descriptions by Howe et al. (2009), Tvardikova and Novotny (2012), Low et al. (2014), and Nurdiansyah et al. (2016). The recorded predation marks were from the predators’ mandibles, ovipositors, beaks, bird's claws, and teeth.

### 2.4 Assessment of environmental variables

We assessed a range of environmental variables to understand their relationship with predation (Table 1). The variables measured were as follows: (i) canopy cover; (ii) plot elevation; (iii) understory vegetation cover; (iv) height of understory vegetation; and (v) proximity to forest. Variables i, ii, iii, and iv were measured within a 10‐m‐radius circle haphazardly located at each plot. We measured canopy cover using Gap Light Analysis Mobile Application (Glama) version 3.0 (Tichý, 2016). This application tool supports the calculation of canopy openness and measures tree layer cover by analyzing a hemispherical photograph. The understory vegetation cover was visually estimated at each plot (Milligan et al., 2016). Understory vegetation cover was divided into the grass and nongrass vegetation. We used Global Positioning System (GPS) to determine elevation at each plot. The distance of each sampling plot to the nearest contiguous forest (>10,000 ha) was measured using Google Earth Pro measuring tools.

**Table 1:** Summary statistics of environmental variables in each land‐use type

Explanatory variable	Mean ± *SD*	Median	Min – Max
Fruit orchard			
Canopy cover (%)	55.40 ± 10.31	56.96	26.29–79.20
Elevation (m)	45.08 ± 12.09	43	28.50–76
Grass coverage (%)	39.60 ± 25.15	35.50	2–81
Grass height (cm)	18.05 ± 19.43	9.95	3.60–96.40
Nongrass coverage (%)	30.40 ± 23.44	21	4–95
Nongrass height (cm)	44.42 ± 31.39	36.30	4.20–193.10
Proximity to forest (km)	5.19 ± 2.97	4.62	0.34–14.11
Oil palm plantation			
Canopy cover (%)	50.76 ± 7.16	52.28	31.83–61.45
Elevation (m)	58.46 ± 15.58	59.70	25.40–98
Grass coverage (%)	40.47 ± 25.08	39	5–90
Grass height (cm)	10.46 ± 6.20	9.50	3.20–32.60
Nongrass coverage (%)	32.13 ± 23.22	25.50	4–91
Nongrass height (cm)	45.40 ± 22.53	42.90	9.50–110.30
Proximity to forest (km)	5.99 ± 2.02	5.49	3.57–9.77
Rubber tree plantation			
Canopy cover (%)	56.36 ± 5.99	55.28	45.10–73.14
Elevation (m)	53.26 ± 18.40	49.85	21.60–96
Grass coverage (%)	43.57 ± 22.96	42.50	1–89
Grass height (cm)	17.34 ± 14.11	13.80	3–80.70
Nongrass coverage (%)	39.13 ± 21.33	39	4–89
Nongrass height (cm)	66.45 ± 39.30	58.65	15.40–168.10
Proximity to forest (km)	7.93 ± 3.66	8.82	3.78–13.45

### 2.5 Statistical analysis

All statistical analyses were conducted in R version 3.6.1 (R Core Team, 2019). We only analyzed data from artificial caterpillars attacked by natural predators and excluded missing artificial caterpillars (Nurdiansyah et al., 2016).

In order to examine whether total predation differed between land‐use types and how this differed between arthropods, mammals, and birds, we fitted generalized linear mixed models (GLMMs) with a binomial distribution with predation as a binary response variable (evidence of predation or not) and habitat type and predator type as interacting categorical predictor variables. We also tested whether environmental variables: (i) canopy cover; (ii) plot elevation; (iii) understory vegetation cover; (iv) height of understory vegetation; and (v) proximity to forest had any effect on predation. We fitted plot nested within plantation number as random effects to account for the nested sampling design, for example, predation ~ habitat type * predator type + (1*|plantation number/plot*). After extensive data exploration and model selection using AIC (Burnham, Anderson, & Huyvaert, 2011), we discounted models with all of the environmental variables as they did not improve model fit and had no noticeable effect on predation.

## 3 RESULTS

### 3.1 Predation of artificial caterpillars

A total of 294 of the 900 artificial caterpillars deployed across the study were identified as being predated. Sixty‐two caterpillars were reported missing from the deployed locations. Predation by arthropods (17.6% = 158 marks) was the highest, followed by mammals (12.3% = 111 marks) and birds (2.8% = 25 marks) (Table 2). Our models showed that there was no difference in the total amount of predation of artificial caterpillars between oil palm plantations, rubber tree plantations, and fruit orchards (Table 3). Arthropods were the most common predators in all three land‐use types with bird and mammal predation 100 times and 15 times less likely than arthropod predation, respectively, according to model estimates (Table 3). Predation by arthropods and birds did not differ between land‐use types; however, mammal predation was 3.6 and 2.6 times more likely in fruit orchards and rubber tree plantations than in oil palm plantations.

**Table 2:** Summary of predation on artificial caterpillars by arthropods, birds, and mammals

Location	Number of predation			
	Arthropod	Bird	Mammal	Overall predation by land use
Fruit orchard	42	6	45	93
Oil palm	65	5	24	94
Rubber	51	14	42	107
Overall predation by taxon	158	25	111	

**Table 3:** GLMMs of caterpillar predation with organism type (arthropods, birds, and mammals) and habitat type (oil palm, orchard, and rubber tree) as interacting predictor variables. Predation by arthropods in oil palm is the intercept of the model of which all other variables are compared to using the odds ratio

Predictors	Odds ratio	CI	*p*
Arthropod	0.23	0.13–0.40	
Bird	0.06	0.03–0.15	<.001
Mammal	0.29	0.17–0.48	<.001
Orchard (arthropod)	0.59	0.26–1.34	.208
Rubber (arthropod)	0.81	0.36–1.80	.601
Bird*Orchard	1.43	0.41–4.98	.572
Mammal*Orchard	3.56	1.80–7.05	<.001
Bird*Rubber	2.44	0.83–7.23	.106
Mammal*Rubber	2.64	1.34–5.17	.005

Note

Symbol “*” denotes a model interaction.

### 3.2 Effect of environmental variables on predation

None of the environmental variables (e.g., distance to forest, understory vegetation height/cover, elevation) had any noticeable effect on predation.

## 4 DISCUSSION

We found that arthropods were the most important predators across all land‐use types, with mammals also providing a significant role. However, birds were minor predators, in all three land‐use types.

Our findings share similarities with previous work that found that arthropods were the primary predator group in oil palm and rubber tree plantations, with mammals and birds more minor predators (Nurdiansyah et al., 2016). However, predation rates by birds and mammals were much higher at our study sites than in the previous study, particularly predation by mammals (>12% of all caterpillars vs. <2%).

Furthermore, although predation rates were broadly similar among land‐use types, in our study, predation by mammals was almost twice as high in fruit orchards and rubber tree plantations than in oil palm plantations. This perhaps reflects a higher abundance of mammals in these land‐use types. Bats and rodents are likely to be the main mammalian predators in this study, as they are common in agricultural habitats in South‐East Asia (Buckle, Chia, Fenn, & Visvalingam, 1997; Maas, Clough, & Tscharntke, 2013; Phommexay, Satasook, Bates, Pearch, & Bumrungsri, 2011; Syafiq et al., 2016). However, oil palm plantations have been found to poorly support insectivorous mammals (Yue, Brodie, Zipkin, & Bernard, 2015). Insectivorous bats are an important predator group that is less likely to be accurately assessed with artificial caterpillar techniques. This is because of their use of echolocation while foraging.

Predation by birds was very low in our study, likely caused by low abundances of insectivorous in the three land‐use types. Insectivorous bird abundance and diversity have been found to be greatly reduced when forest habitats are converted to oil palm plantations and rubber tree plantations, in particular (Azhar et al., 2011; Prabowo et al., 2016; Srinivas & Koh, 2016).

Our study showed that both arthropods and mammals play important functional roles as predators and consequently may provide important pest control ecosystem services in tropical agricultural landscapes. Interestingly, none of the environmental factors we measured had appreciable effects on rates of predation. This could be due to our fine‐scale sampling approach of environmental variables compared to the relatively heterogeneous management practices between plantations and fruit orchards across our study area. All land‐use types were owned and managed by smallholders, and therefore, trees were managed differently and planted at different times. This heterogeneity, in combination with the small size of the farms, may have confounded any relationship between environmental variables (e.g., understory vegetation cover and canopy cover) and predation rate. Stronger relationships of environmental variables (e.g., understory vegetation structure) with ecosystem function may be found in larger‐scale monoculture plantations where medium‐scale heterogeneity is low (Ashton‐Butt et al., 2018).

Nevertheless, understory vegetation can provide habitat and food plants for predatory arthropods (Ashraf et al., 2018; Spear et al., 2018; Tews et al., 2004). Therefore, reducing herbicide use and allowing understory vegetation to proliferate could be an important management tool, in all three land‐use types, where arthropods were by far the most significant predator. Predation by mammals was higher in fruit orchards and rubber tree than in oil palm plantations. Lower predation rates likely reflect the poor ability of oil palm to support insectivorous mammals (Yue et al., 2015). While higher predation in fruit orchards is likely to be related to polyculture farming systems which includes a mix of native tree species and crops such as banana and mangoes attracting small mammals like bats (Syafiq et al., 2016). In rubber tree plantations, the higher predation by mammals could be due to the mix of perennial fruit tree species planted alongside rubber trees. Commercial crop production landscapes with more heterogeneous vegetation for the provision of refuges can increase mammal diversity (Ramírez & Simonetti, 2011) and in turn enhance ecosystem service provision (Landis, 2017).

Our study provides useful experimental results which would be difficult to obtain through alternative, indirect methods (Howe et al., 2009); nevertheless, there are a number of limitations associated with the use of artificial caterpillars which may confound our results. Ideally, some sort of comparison with living organisms would be useful to validate how effectively the artificial caterpillars simulated real living organisms.

Differences in predation between taxa were found; in particular, predation by birds was low in our study. The lack of predation by birds could be the result of a number of reasons. First, bird abundance may be low as insectivorous birds are often adversely affected by the conversion of forest to agricultural land use, particularly in oil palm (Azhar et al., 2011; Srinivas & Koh, 2016). Alternatively, insectivorous birds can also be very selective in their diet (Morse, 1971) and may not have been attracted to the artificial caterpillars, in our study system, thus biasing the results. However, this is unlikely, as birds have been identified as the main predator in previous studies using artificial caterpillars (Maas et al., 2015).

Our results may not reflect the canopy level of each land‐use type. Deploying the artificial caterpillars at the canopy level is impractical. High air temperature at the canopy level may damage the artificial caterpillars. Another methodological uncertainty is the disappearance of artificial caterpillars during the study. This could have been caused by weather conditions (e.g., heavy rain) or complete consumption by large mammals (e.g., cattle and buffalo). There were also signs that long‐tailed macaque (*Macaca fascicularis)* detached some of the deployed artificial caterpillars.

## 5 CONCLUSION

Our study demonstrates that arthropods and mammals play an important functional role as biological control agents in the understory of oil palm plantations, rubber tree plantations, and fruit orchards. To support sustainable agricultural management, the diversity and abundance of arthropod and mammal predators should be maintained by tolerating understory vegetation and minimizing application of pesticides. Predation by birds was very low in our study, likely reflecting the poor ability of the three land‐use types to support insectivorous birds. While further research is necessary to characterize the economic value of beneficial ecosystem services provided by natural predator populations in oil palm plantations, rubber tree plantations, and fruit orchards, we show that arthropods and mammals, in particular, are important predators and thus management efforts should be made to conserve beneficial arthropod and mammal diversity and abundance.

## CONFLICT OF INTEREST

The authors declare that they have no conflict of interest.

## AUTHOR CONTRIBUTIONS

N.D., N.R., R.S., and B.A. conceived the ideas; N.D. and B.A. analyzed the data; and N.D., W.Z.W.M., D.M.N., N.R., R.S., F.N., A.A., A.L., and B.A. wrote the article.

## Data Availability

Empirical data have been archived in DataDryad: https://doi.org/10.5061/dryad.8sf7m0chc.
